# A wireless, implantable sensor for continuous monitoring of blood leakage after endovascular aneurysm repair

**DOI:** 10.1126/sciadv.ady6148

**Published:** 2025-10-01

**Authors:** Sun Young Park, Soo Hyun Kim, Unseong Baik, Seung Jae Huh, Raudel Avila, Hyeon Ho Shin, Hyoungsuk Yoo, Heungsoo Shin, Jin-Tae Kim, Young-Hyo Lim, Yei Hwan Jung

**Affiliations:** ^1^ Department of Electronic Engineering, Hanyang University, Seoul 04763, Republic of Korea.; ^2^ Department of Artificial Intelligence Semiconductor Engineering, Hanyang University, Seoul 04763, Republic of Korea.; ^3^ Department of Mechanical Engineering, POSTECH, Pohang 37673, Republic of Korea.; ^4^ Department of Bioengineering, Hanyang University, Seoul 04763, Republic of Korea.; ^5^ Department of Mechanical Engineering, Rice University, Houston, TX 77005, USA.; ^6^ Division of Cardiology, Department of Internal Medicine, College of Medicine, Hanyang University, Seoul 04763, Republic of Korea.; ^7^ Institute of Nano Science and Technology, Hanyang University, Seoul 04763, Republic of Korea.

## Abstract

Endovascular aneurysm repair, a treatment for abdominal aortic aneurysms, carries the risk of recurrence due to endoleaks following stent graft implantation. Since these leaks do not present with specific symptoms, regular follow-up is necessary; however, now, only imaging-based monitoring is available. This study proposes an ultrathin flexible sensor inserted endovascularly with a stent to detect type I endoleaks, which pose the highest rupture risk. A coplanar capacitive sensor was attached to the stent graft using a specially developed flexible thermal adhesive to monitor blood leakage, without compromising the stent graft’s function. It seamlessly folds and unfolds with the stent, allowing implantation through catheter-based procedures. Experimental validation confirmed that the sensor does not induce blood leakage and demonstrated long-term stability and functionality under vascular and dynamic conditions. This technology enables wireless monitoring of endoleak status, facilitates timely intervention, and improves postoperative outcomes by reducing the risk of recurrent aneurysms.

## Teaser

The thin flexible sensor integrated into the stent monitors blood leakage wirelessly, enabling early identification of endoleak.

## INTRODUCTION

Endovascular aneurysm repair (EVAR) (*1*) is a minimally invasive technique for treating abdominal aortic aneurysms by directing blood flow through a stent graft implanted within the weakened aortic wall (*2*, *3*). Compared with traditional open surgery, EVAR offers considerable advantages, including shorter recovery time and lower complication rates (*4*–*6*). However, one of the most critical concerns after EVAR is the occurrence of endoleaks (*7*), which can lead to continued aneurysm expansion, increased pressure, and potentially severe complications such as aneurysmal ruptures or circulatory impairments (*8*). Type I endoleaks, which arise from incomplete sealing of the stent graft at the proximal or distal attachment sites (*9*–*12*), present the highest risk of rupture and mortality (*9*, *13*). Clinical studies indicate that type I endoleaks occur in ~3% of patients with EVAR, with most cases requiring immediate intervention because of their association with persistent sac pressurization and aneurysmal rupture (*14*). Without timely treatment, type I endoleaks can lead to aneurysmal rupture within a median period of 11 months, with some cases progressing earlier than others (*11*). Mortality rates in patients with unresolved type I endoleaks are quite high, with in-hospital mortality reported to be ~27.3% (*15*). These findings underscore the critical need for early detection and prompt intervention to mitigate life-threatening complications (*16*). The proposed real-time monitoring sensor system offers a significant technological advantage by enabling near-continuous post-EVAR surveillance, in contrast to conventional imaging-based follow-up protocols, which typically assess the endoleak presence at 1, 6, and 12 months postoperatively and annually thereafter. This system allows patients to perform routine self-monitoring, facilitating early detection of physiological changes indicative of endoleak development. In addition, by identifying the position of leakage from the anterior or posterior vessel wall, the system can assist in selecting the appropriate device, determining the optimal access route and improving procedural strategies for secondary interventions such as stent expansion or cuff insertion. Now, no sensors are coimplanted with stent grafts for endoleak detection, and conventional monitoring relies on periodic imaging techniques such as computed tomography (CT) angiography or magnetic resonance imaging (MRI) (*17*–*21*). CT remains the most commonly used clinical imaging modality for endoleak detection; however, it involves exposure to ionizing radiation and the use of nephrotoxic contrast agents, which pose serious risks, particularly in patients with impaired renal function. MRI provides a safer alternative with respect to radiation exposure and contrast safety, but it is often limited by lower accessibility, higher cost, and insufficient spatial resolution—especially when detecting small or slow-flow endoleaks (*22*–*24*). Follow-up imaging is typically performed at intervals ranging from 6 weeks to 3 months, during which time an endoleak may go undetected, allowing for silent progression of the aneurysm (*25*, *26*). Diagnosis often relies on monitoring changes in aneurysm sac diameter over time, with sac enlargement considered a key indicator of endoleak-related complications. However, sac expansion represents a relatively late sign, and sole reliance on this parameter may delay timely diagnosis and therapeutic intervention (*21*, *27*).

Moreover, detecting minor blood leakage at the proximal attachment site of the stent graft presents considerable engineering challenges, including high-intravascular pressure, flexible and dynamic movement of vascular walls, and continuous biomechanical forces from pulsatile blood flow. To address these limitations, this study introduces a wireless, real-time blood leakage monitoring technology that enables the continuous detection of type I endoleaks. The proposed system integrates an ultrathin capacitive sensor (*28*, *29*) array in a filamentary serpentine configuration coupled with an inductor-capacitor (LC) resonant circuit, which is seamlessly embedded within the proximal attachment site of a conventional stent graft. The sensor is positioned between the stent graft and the vessel wall, where it provides precise, localized information on blood flow and leakage based on capacitive principles that differentiate between the blood and the vessel wall. The ultrathin and flexible nature of the sensor ensured that it did not interfere with the mechanical performance of the stent and maintained its structural integrity even after repeated cycles of crimping, folding, and deployment. The sensor remains imperceptible to blood flow, preserving the primary function of EVAR while simultaneously detecting potential leaks before they become clinically important. Blood leakage results in detectable shifts in the capacitance, which are reflected in the resonance frequency changes within the LC circuit. These frequency variations can be wirelessly detected via inductive coupling to an external reader positioned on the patient’s abdomen, providing continuous and noninvasive monitoring of the endoleak status (*30*). Computational simulations, experimental validation using blood samples, electrode cytotoxicity assessments, and in vitro studies using a porcine aorta model confirmed the feasibility of this technology for clinical applications. By enabling real-time endoleak detection, this system has the potential to revolutionize post-EVAR monitoring, reduce the risk of aneurysm rupture, and improve patient outcomes.

## RESULTS

### Device design, functionality, and underlying concept

**Fig. 1.**
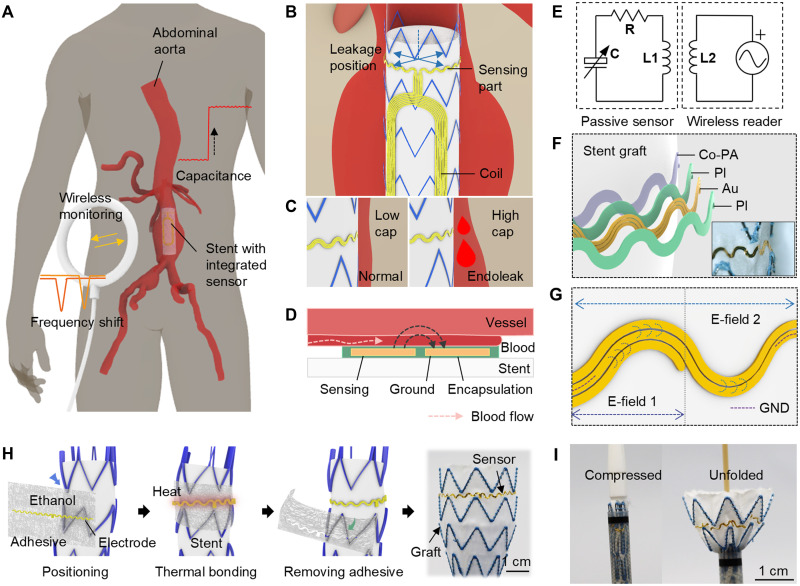
Overview of a capacitance electrode sensor integrated into the stent and the principle of endoleak detection. (**A**) Schematic illustration of the endoleak detection sensor installed on a stent within an aortic aneurysm demonstrating a wireless endoleak detection mechanism using capacitance-based resonant frequency sweeps. (**B**) Schematic of the sensor configuration for position detection, with four sensors positioned along the circumference of the stent to detect its orientation. (**C**) Increased capacitance owing to endoleaks. Left: Normal conditions leading to low capacitance. Right: Conditions illustrating blood leakage leading to increased capacitance. (**D**) Schematic illustration showing cross-sectional view of the electrode between the vessel and the stent and the electric field distribution of the capacitive sensor. (**E**) Circuit diagram of the LC-based wireless system. R, resistor; C, capacitor. (**F**) Exploded schematic illustration showing the structure of the electrode sensor attached to the graft surface using a thermal adhesive. Inset shows a photograph of the sensor mounted on the outer surface of the stent. (**G**) Schematic of sensor configuration for position detection. GND, ground. (**H**) Three-dimensional schematic representation of the thin electrode transferred onto the stent using a thermal adhesive. (**I**) Photographs of the compressed stent with electrodes loaded onto the catheter and the stent in its unfolded state.

Figure 1A illustrates the conceptual medical application of this blood leakage monitor in patients implanted with an EVAR stent. In this concept, the device facilitates the noninvasive and wireless monitoring of potential blood leaks using an external reader. The electrode specifically targets type Ia endoleaks that occur at the upper proximal end of the stent owing to vascular degeneration or stent migration (*31*). This condition poses the greatest risk among endoleak types owing to the high-pressure environment within the aneurysm, lack of spontaneous resolution, and the necessity for immediate surgical intervention. In stent graft procedures used to treat aneurysms, the proximal end of the stent must overlap the normal aortic region by at least 20 mm (*32*, *33*). This region represents a critical site of seal failure due to endoleak, and positioning the electrode at the proximal end enables effective monitoring of this vulnerable area, thereby facilitating early detection and timely clinical intervention. Therefore, installing a capacitive sensor in the space below the first shape memory alloy is the most effective method for detecting blood leakage. As shown in Fig. 1B, the EVAR stent, with an average proximal diameter of 28 mm and a tubular length of 150 mm (*34*), incorporates a circumferential ring of capacitive leakage sensors at the upper end for blood leakage monitoring and features a large inductive coil along its length to enhance the coupling efficiency with an external reader. The attachment orientation of the coil was referenced to the radiopaque marker used in the stent graft, facilitating the interpretation of directional signals after implantation (fig. S1) (*35*, *36*). As shown in Fig. 1 (C and D), blood leakage at the proximal end causes a capacitance change in the sensor, shifting the resonant frequency of the connected inductive coil, which can be continuously monitored wirelessly using an external coil system placed near the abdominal skin (Fig. 1E). Figure 1F shows that the device consists of gold electrodes encased in a biocompatible polyimide (PI). The thin and flexible structure of the device (total thickness of 5 μm; fig. S2) allowed seamless integration with the stent during aortic stent placement, thereby eliminating the need for additional surgical procedures. The sensor can be integrated during the semimanual fabrication process of the stent graft, which is customized to match the patient’s vascular anatomy (*37*–*39*). The coplanar capacitor electrodes surrounding the stent generated electric fields with four electrode pairs for spatiotemporal identification of leakage. As shown in Fig. 1G, depending on the location of the blood leakage, capacitance changes occur either in a single region of the sensor or in both regions. Four distinct electrode pairs are positioned circumferentially around the proximal region of the stent, enabling localization of the leakage site through detection of capacitance change upon contact with blood. This serpentine structure expands the capacitance region and increases the bonding area, thereby enhancing adhesion stability (fig. S3). In addition, this design minimized the effective Young’s modulus of the structure, allowing for relatively unrestricted folding and deployment during catheter-based implantation procedures (*40*, *41*).

The device was securely attached to the stent graft using a specially formulated biocompatible adhesive to ensure robust bonding with the graft fabric material. The adhesive was made by dissolving co-polyamide (Co-PA) material in a solution of mixed tetrahydrofuran (THF) and phosphate-buffered saline (PBS), which was then fabricated into a film form. As shown in Fig. 1H, the process of transferring the microscale thin and flexible electrodes onto the stent fabric involved adhering the device to an adhesive film. The adhesive film was prepared by spin-casting a heated adhesive onto a silicon wafer, which was then separated from the wafer. Ethanol facilitates the initial adhesion between the adhesive and the stent (*42*), which is subsequently reinforced through thermal bonding at 110°C. The synthesized adhesive exhibited excellent mechanical and adhesive properties, maintaining its structural integrity and functional stability in dynamic vascular environments. Figure 1I illustrates the mechanical performance of the thin, flexible adhesive, showing that the integrated sensor did not compromise the self-expanding functionality of the stent. Specifically, the sensor, integrated with the stent and deployed using a commercial catheter, maintained secure adhesion to the stent fabric without any damage or detachment even after repeated compression and expansion deformations. As a result, the combination of a thin, chipless sensor system and flexible adhesive has proven to be crucial for maintaining a stable contact with the vessel and enabling long-term endoleak detection, even in the aorta—the region with the largest diameter and highest blood pressure—where surgical incision is not possible.

### Sensor performance to blood leakage

The coplanar capacitive sensor applied to the stent consisted of two electrodes, an anode and a cathode, to measure changes in relative permittivity of the medium that is in contact with the sensor. Various environments, including air, blood, vessels, fat, and albumin, were analyzed using the sensor across a frequency range of 0 to 200 kHz (fig. S4). All samples demonstrated linear capacitance behavior within the 50- to 200-kHz range. Specifically, at 100 kHz, blood (19.8 pF) exhibited the highest capacitance, followed by albumin (16.3 pF), vessels (13.8 pF), and fat (1.7 pF), in descending order. In addition, phantom models of blood and vessels were developed and calibrated to correlate them with actual samples to conduct a feasibility analysis on the sensor (fig. S5). The capacitance values of the blood and vessel phantoms were consistent with those of the actual materials over the entire range of 0 to 200 kHz, thus confirming their suitability for quantitative experimental assessments.

**Fig. 2.**
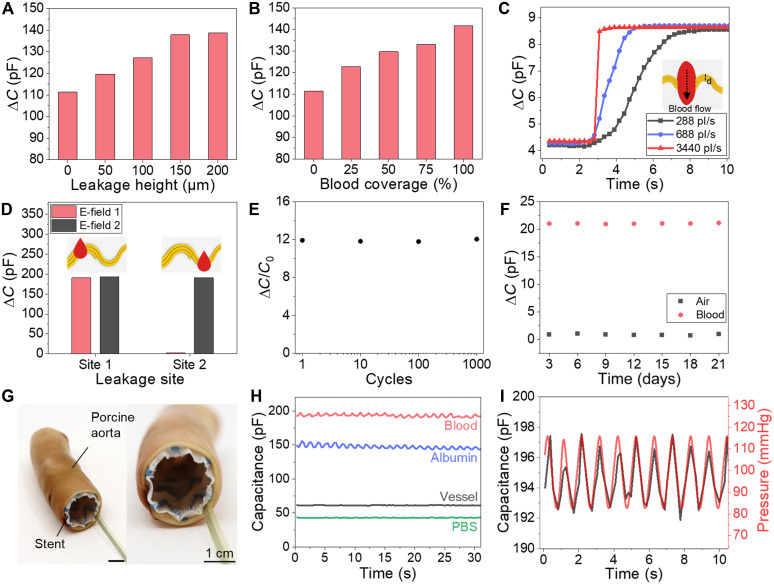
Sensitivity and reliability of the electrode sensor and ex vivo experimental outcomes of the sensor integrated into the stent. (**A**) Capacitance data according to blood thickness. (**B**) Capacitance data according to the blood-covered area (electrode length: 25 mm). (**C**) Graph depicting the sensor’s temporal response at different initial blood flow velocities. (**D**) Capacitance data identifying the site of blood leakage: Blood flow at site 1 causes increased capacitance at both electric field (E-field) 1 and 2, while blood flow only at site 2 increases capacitance solely at E-field 2. (**E**) Graph showing data consistency after 1000 cycles of compression and expansion stress testing. (**F**) Graph showing daily data for the sensor submerged in PBS for 1 month. (**G**) Photograph depicting the ex vivo setup using a porcine aorta. (**H**) Capacitance data recorded under the conditions depicted in (G) when blood, albumin, and PBS flowed between the stent and porcine aorta. (**I**) Data variation observed within the normal physiological blood pressure range.

Figure 2A shows the sensitivity and functionality of the sensor for accurate detection of the volume of leaked blood by measuring the leakage area of blood in contact with the sensor. In particular, the capacitance increased linearly with the area of blood leakage. In Fig. 2A, it can be observed that capacitance increases as the blood thickness increases at 0, 50, 100, 150, and 200 μm. The sensor can detect blood thicknesses up to 150.25 μm based on the coplanar capacitor principle, where the electric field propagates from the anode to the cathode in a parabolic trajectory through the target medium (*43*). This limitation could potentially be overcome by increasing the anode-to-cathode distance; however, as blood leakage exceeding 150 μm is indicative of total leakage, such an adjustment is unnecessary (*44*). In addition, we have confirmed that blood detection remains possible even in the presence of a fibrin sheath, which typically forms very thin layers ranging from a few nanometers to tens of nanometers (fig. S6) (*45*–*47*). In addition, increasing the distance may reduce sensitivity owing to the influence of flowing arterial blood along the inner wall of the stent graft. As shown by the measured results in fig. S7, the sensor was not affected by blood flow inside the stent. Figure 2B shows that as the lateral coverage of blood (i.e., width of blood leakage) infiltrating between the vessel and the electrode per sensor increased due to blood leakage, the capacitance also increased. The sensor can also measure the initial leakage velocity by monitoring capacitance changes over time. Figure 2C demonstrates the capacitance variation from the onset to the equilibrium stage of blood leakage, with a stationary volume of 1000 mm^3^ above the sensor at three different blood flow rates (288, 688, and 3440 pl/s). The resulting capacitance-time plot shows that as the blood flow rate increases, the rise time of the capacitance decreases, providing key insights for assessing the urgency of leakage based on the flow rate. Figure 2D shows that the leakage site can be detected by analyzing the capacitance values generated by the electric field of the electrode lines. Depending on the location of the blood leakage, capacitance changes occur in either a single region or in both regions of the sensor. As the severity of leakage increases, the corresponding changes in capacitance become more pronounced, enabling effective monitoring of capacitance variations under both single-site and multi-site leakage conditions (fig. S8). Figure 2E shows that the capacitance of the electrode attached to the stent remained stable even after more than 1000 cycles of stent compression and expansion using a stent-crimping machine. This indicates the capacity of the sensor for long-term, stable detection in the dynamic environment of the aorta, which experiences high-blood pressure even after postimplantation (figs. S9 and S10).

### Ex vivo evaluation of sensor in a porcine aorta model

Figure 2F shows the long-term stability of the sensor in an ex vivo environment. The sensor was immersed in a PBS solution at 37°C, and its capacitance response was measured over 1 month, without any notable capacitance change. Native capacitance (air) and blood detection values were measured at 3-day intervals, showing repeatable outcomes and suggesting the potential of the sensor for long-term in vivo applications.

Figure 2G shows the results of an ex vivo study using a porcine aorta model, demonstrating the feasibility of the sensor as an endoleak monitor. The leakage environment between the porcine aorta and the upper part of the stent was replicated on a bench top setup. In this setup, a stent with an attached sensor was inserted into the porcine aorta via a catheter, and blood was injected between the aorta and stent to mimic a type 1a endoleak. To replicate the arterial blood pressure, repetitive pressures ranging from 80 to 120 mmHg at a rate of 80 to 90 beats per minute (bpm) (*48*) were applied to the stent via internal air pressure (fig. S11). Figure 2H shows the changes in the capacitance values for different materials under ex vivo conditions. Capacitance changes were observed when blood vessels, albumin, and PBS were injected between the vessel and the stent using a syringe, and consistent values were maintained for each material, even during sequential cross-injections. In terms of the electrical response, the sensor exhibited the highest capacitance in blood, followed by albumin and PBS. Arterial pressure fluctuations at a normal heart rate can induce subtle variations in both vessel wall thickness and the presence of leaked blood. Unlike PBS, which exhibits Newtonian behavior with constant viscosity, blood is a non-Newtonian fluid comprising various cellular components, resulting in more pronounced fluctuations in response to changes in arterial pressure (fig. S12). These minute changes result in corresponding variations in capacitance, as detected by a coplanar capacitor sensor interfaced with the surrounding medium. The sensor registers minute capacitance changes within this physiological pressure range. This dual sensitivity enables simultaneous monitoring of the heart rate and detection of potential blood leakage. Figure 2I compares the capacitance changes measured by the sensor (black line) with the pressure values reproduced by the pump within the blood vessel (red line). Capacitance fluctuations corresponding to 6.82% of the leak-induced increase were observed under physiological pressure conditions (80 to 120 mmHg). Even under hypertensive conditions (100 to 180 mmHg), the fluctuation remained within 25.13% of the leak-induced increase. These results demonstrate that the effect of blood pressure-induced mechanical disturbances on the sensing signal is well controlled, thereby enabling accurate and reliable heart rate monitoring (fig. S13).

### Thermal adhesive for sensor attachment

The EVAR stent graft is a fabric-like waterproof material, such as Gore-Tex, providing flexibility and excellent mechanical stability, allowing it to expand naturally and conform to the blood vessel. The adhesive used for bonding electrodes to graft surfaces in intravascular applications must have the following key properties: (i) mechanical flexibility comparable to that of the stent fabric, (ii) strong adhesion for long-term implantation, (iii) controlled curing to prevent inner wall penetration and circulatory interference, and (iv) high biocompatibility to minimize the inflammatory response. These characteristics help mitigate functional damage to the electrodes, reduce the risk of detachment, and minimize the potential for an immune response. Conventional adhesives have limitations that render them unsuitable for attaching ultrathin devices to stent graft materials. For example, existing adhesives for biomedical applications, such as tissue adhesive medical glues, exhibit flexibility but undergo biodegradation in vivo, limiting their suitability for long-term implantation (*49*). Conversely, bone adhesives provide long-term stability but have high rigidity, making them unsuitable for vascular applications (*50*). The thermal adhesive proposed in this study overcomes the limitations of existing adhesives and enables stable functioning even in intravascular environments. We developed an adhesive suitable for stents using materials that harden with heat but maintain flexibility, which are commonly used for fabric bonding. This adhesive combines mechanical flexibility, strong adhesion for long-term implantation, controlled curing to prevent inner-wall penetration, and high biocompatibility.

**Fig. 3.**
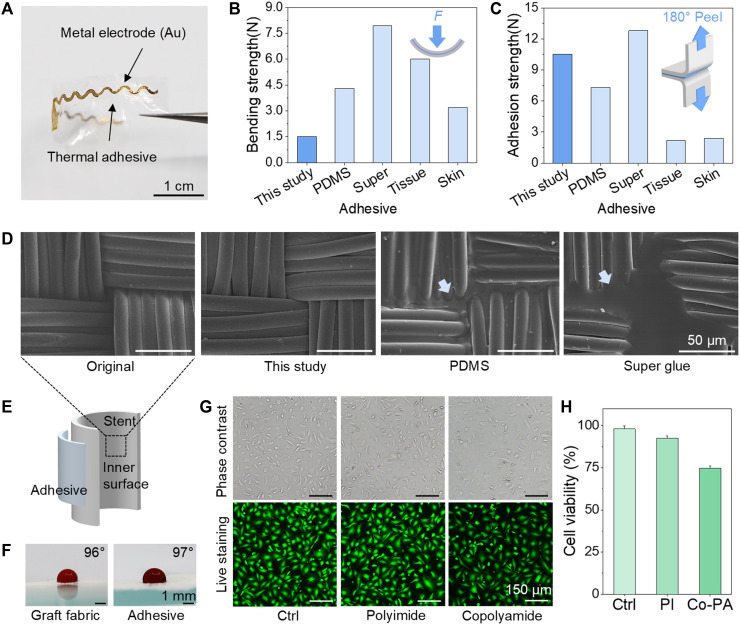
Characteristics of the developed thermal adhesive. (**A**) Photographs of the thermal adhesive and electrodes. (**B**) Graph showing the bending strengths of the tested adhesives after curing to demonstrate the flexibility and (**C**) adhesion strengths of the adhesives. (**D**) SEM images of the inner surface of the stent fabric after coating or attaching the adhesive on the outer layer, followed by full curing, illustrating the degree of adhesive penetrations. (**E**) Schematic depicting the application of adhesive to the outer wall of the stent. (**F**) Contact angles of blood droplets on the inner graft fabric after adhesive application. (**G** and **H**) Calcein staining and cell viability analysis.

The adhesive exhibited flexibility, strong adhesion, and biostability, making it highly effective for bonding microscale thin film devices to graft materials (Fig. 3A). Figure 3 (B and C) compares the bending and adhesion strengths of the developed adhesive with those of various industry-standard adhesives, including polydimethylsiloxane (PDMS), a medical liquid super-adhesive (ferndale mastisol adhesive 52348), a tissue adhesive (3M vetbond), and a topical skin adhesive (skinister topical skin adhesive). An ideal adhesive material should have low-bending strength and high-adhesion strength. The thermal adhesive demonstrated a low-bending strength of 1.5 N, reflecting its flexibility while achieving a high-adhesive force of 10 N. In comparison, PDMS exhibited a higher bending strength (4.5 N) but a lower adhesive force (6.1 N), whereas super glue showed the highest bending strength (7.6 N) and adhesive force (12.8 N). Tissue glue and skin glue displayed greater bending strengths and lower adhesive forces than the developed adhesive (tissue glue: bending strength = 6.0 N, adhesive force = 2.2 N; skin glue: bending strength = 3.2 N, adhesive force = 2.4 N). Peel tests confirmed consistent adhesive strength under prolonged physiological conditions, with no reduction in thickness after extended PBS immersion. Electrodes bonded to the stent also showed no delamination or damage after 14 days in 95°C PBS, demonstrating both hydrolytic and thermal stability of the adhesive interface (fig. S14). Following mechanical fatigue testing to evaluate the durability of the adhesive, no evidence of cracking or delamination was observed on the electrode surface, demonstrating its outstanding mechanical stability (fig. S15). These findings highlight the fact that the developed adhesive has an optimal combination of properties suitable for implantation in high-pressure blood vessels.

Complete curing of the adhesive must ensure that it does not penetrate the surface of the stent graft as possible penetration into the inner tube could compromise blood flow. To assess adhesive penetration, the inner wall of the stent was examined using scanning electron microscopy (SEM) after applying the adhesive to its outer surface (Fig. 3, D and E). As a result, the developed adhesive did not penetrate the stent inner wall, and no notable changes were observed on the inner wall compared to the untreated stent. Upon thermal activation, the polyester matrix infiltrated the microscale gaps and pores between individual fabric filaments, forming a mechanically interlocked structure through physical entanglement with the fabric network (fig. S15). This behavior is attributed to the initial film form of the adhesive, which allows for precise control over the applied material by adjusting the film thickness. The adhesive film used had a thickness of 25 μm, which was optimized to provide the desired adhesion properties while preventing any excess material from penetrating the interior parts. In addition, the rapid curing duration of 5 s at 110°C minimizes the risk of penetrating the fabric material during the curing process. In contrast, SEM analysis exhibited a minor penetration of PDMS and cyanoacrylate (super glue) into the graft material, which can be attributed to their liquid states, characterized by low viscosities and extended curing times (*51*). Figure 3F shows the assessment of the hydrophobicity of the inner surface of the graft fabric following adhesive application, confirming that the integration of sensors with the adhesive does not compromise the intrinsic waterproof properties of the material. The contact angle measurements were 96° for the untreated graft fabric and 97° for the adhesive-treated surface, indicating that the hydrophobicity of the stent fabric is maintained after adhesive integration (fig. S16) (*52*). Furthermore, when blood was allowed to flow along the inner surface of the graft fabric, no capacitive signal was detected from the outer surface. These findings indicate that the developed adhesive fulfills the mechanical requirements for securely transferring electrodes onto the outer surface of the stent graft while exhibiting excellent flexibility, strong adhesion, and resistance to penetration.

Biocompatibility assessments of the PI and adhesive demonstrated that the materials used in this study posed no significant cytotoxicity to the endothelial cells (*53*, *54*). Phase-contrast and live-cell imaging of human umbilical cord endothelial cells (HUVECs) cultured in conditioned media containing extracts from each material showed comparable cell densities and sprouting tendencies across all groups, including the control (Fig. 3G). The quantitative thiazolyl blue tetrazolium (MTT) assay results showed no detrimental effects of the conditioned media from PI film on HUVECs. In addition, the viability outcomes were 92 and 75% in the conditioned media from the PI film and adhesive, respectively (Fig. 3H). The relatively lower live cell percentage observed with adhesive exposure is likely attributable to small-molecule–induced cytotoxicity caused by the imperfectly controlled adhesive manufacturing environment, although this reduction was moderate.

### Numerical, analytical, and experimental evaluation of leakage induced by electrode thickness

**Fig. 4.**
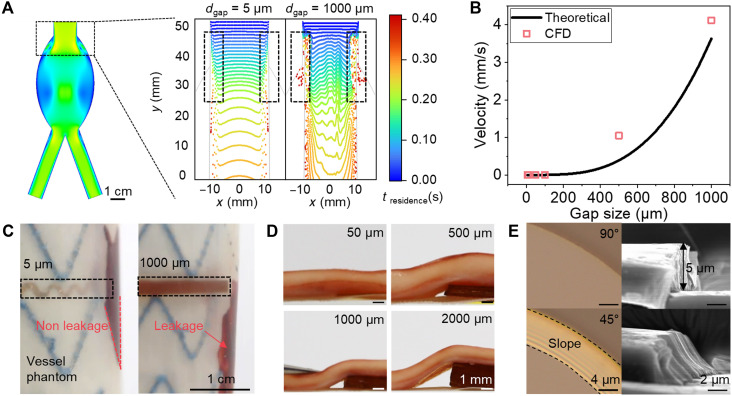
Amount of blood leakage depending on the thickness of the device integrated into the stent. (**A**) Numerical simulation of an aortic aneurysm without stent implantation with velocity magnitude contour (left) and endoleak between the stent and vessel for electrode thicknesses of 5 and 1000 μm with color indicating the residence time of flow particles (right). (**B**) Comparison of numerical simulations with the analytical approach for the velocity of blood leakage as a function of the gap size induced by varying electrode thickness. CFD, computational fluid dynamic. (**C**) Photographs of blood leakage induced by electrodes (thicknesses = 5 and 1000 μm) installed between the vascular phantom and the stent. (**D**) Gap formation between the stent graft and the vessel depending on the device thickness. The gap size increases as the device thickness increases. (**E**) Optical microscope images of PI etched with O_2_ (top) and with a mixture of O_2_ and CF_4_ (bottom) in the etching process.

Figure 4 (A and B) presents the results of numerical simulations based on the Carreau viscosity model (*55*) alongside analytical modeling to predict blood leakage induced by varying electrode thickness. Simulations were conducted for six different gap sizes that can arise from thick sensors: 5, 10, 50, 100, 500, and 1000 μm, under boundary conditions representative of healthy human cardiac characteristics, including a pulsatile frequency of 120 bpm and an average aortic pressure of 100 mmHg. The comparison of blood leakage velocities between the 5- and 1000-μm gaps highlights the critical impact of electrode thickness. In the 5-μm gap case, the blood leakage velocity was less than 10^−3^ mm/s, whereas in the 1000-μm gap case, the velocity exceeded 4 mm/s, an increase that exceeds three orders of magnitude (Fig. 4A). Notably, the large gap caused the formation of a recirculation zone between the electrode inlet and the arterial wall. This recirculation increases the residence time of the flowing particles, potentially elevating the risk of endoleaks. An analytical model was developed to validate the computational results and optimize the device design. The analytical predictions of the gap velocity were in good agreement with the numerical simulations (Fig. 4B). For the detailed computational and analytical methods, please refer to Supplementary Text (fig. S17).

Figure 4C shows the setup of an experiment conducted to verify the simulation results presented in Fig. 4 (A and B) in an actual environment. To observe leakage caused by gaps, a stent integrated with devices with thicknesses of 5 and 1000 μm was fabricated and placed in a cylindrical phantom mimicking the elastic modulus of vascular tissue (Fig. 4D) (*56*, *57*). The device with a thickness of 1000 μm exhibited blood leakage through the gap caused by its thickness, consistent with the simulation results. In contrast, the sensor with a thickness of 5 μm demonstrated reliable performance as it did not cause additional leakage and allowed the metal wire of the stent to block blood flow effectively. The PI encapsulation layer, which constituted ~93.6% of the sensor thickness, insulated the electrodes of the capacitive sensor. It has been demonstrated that an additional thickness of 5 μm does not induce further blood leakage; however, vertically fabricated encapsulation edges are not suitable for achieving a complete seal. To ensure a full seal on the 5-μm-high structure in close contact with the vessel, a uniform 45° slope was applied across the encapsulation edge (Fig. 4E).

### LC-based wireless monitoring for endoleak detection

**Fig. 5.**
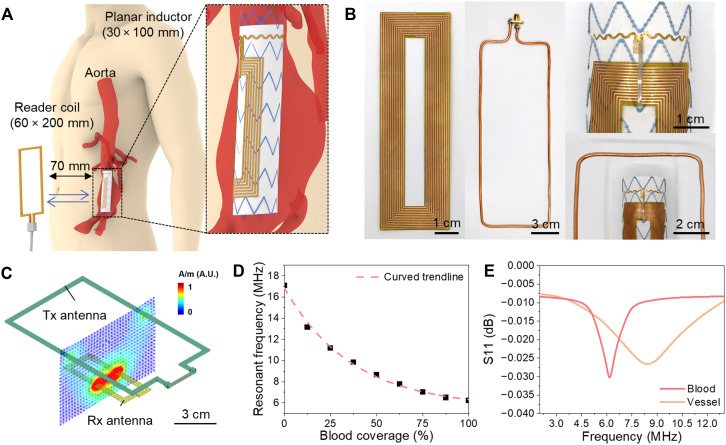
LC-based wireless monitoring system used for endoleak detections. (**A**) Schematic illustration of the LC-based wireless monitoring system designed for endoleak detection applied to the human body. (**B**) Photographs of the planar inductor, reader coil, and electrode sensor integrated into the stent with a planar inductor. (**C**) Simulation setup used to optimize the dimensions of the planar inductor and reader coil. To achieve longer distances, the reader coil (Tx) was designed to be twice the length of the planar inductor (Rx). (**D**) Plot of resonance frequency changes as a function of blood coverage. (**E**) Plot of resonance frequencies measured by placing the vessel and blood on the electrode sensor with a 70-mm gap between the inductor and reader coil. A.U., arbitrary units.

Various wireless systems can be applied to monitor endoleaks externally using electrode sensors attached to a stent. However, the specific environment of insertion within blood vessels requires consideration of factors such as the thickness of the device (*58*). Using an LC-based wireless system in which the inductor of the LC resonant circuit is coupled with a physically separated coil to observe the resonance frequency (*30*), additional data transmission or charging devices are unnecessary, making it extensively applicable for implantable sensors (*59*). As shown in Fig. 5A, the aorta is located deep within the body, typically at a depth of at least 60 mm from the abdominal skin surface (*60*), which poses substantial challenges for wireless data transmission to an external reader. Effective communication at this depth necessitates the use of large inductive coils on both the internal transmitter and external receiver sides. The large surface area offered by the stent graft allows for the integration of a sizable inductive coil on the outer surface of the graft wall. Notably, the metallic structure of the stent does not impede the inductive coupling between the inductor and the external reader coil (fig. S18). To assess the impact of the stent metal on inductive coupling, we measured the S11 parameter of the LC wireless system placed on both a styrofoam block (air-like reference) and the nitinol stent. The resonance frequency showed a negligible shift from 25.5 to 25.1 MHz, with comparable S11 magnitudes, indicating minimal interference. As the stent lies outside the primary coupling path (Fig. 1B) and nitinol exhibits low conductivity (~1/50 that of copper), eddy current generation is minimal (*61*). These results confirm the feasibility of stable wireless sensing with an inductor-integrated stent. This configuration facilitates wireless transmission of capacitance data to an external receiver via an LC resonant circuit. Figure 5B shows the full application of the developed electrode sensor and LC-based wireless system, where the sensor functions as a variable capacitor. The passive sensor consists of an electrode sensor and a planar inductor. The electrode sensor is fabricated by depositing copper (thickness = 200 nm) using an electron beam evaporator, and the planar inductor (30 mm by 100 mm) is prepared using a copper film patterned with a laser ablation system. The wireless reading component consists of a network analyzer to measure the S-parameters and a reader coil. The reader coil (60 mm by 200 mm) has the same shape as the planar inductor but is twice as large and fabricated with a copper wire to facilitate long-range electromagnetic coupling (*62*, *63*). When the planar inductor attached to the stent is aligned with the reader coil, the resonant frequency peak becomes most pronounced (*64*, *65*), allowing for easy detection of resonant frequency changes caused by blood leakage. To confirm whether the sensing capability remained unchanged after integrating the planar inductor into the wireless system, blood was applied in different coverage areas over the electrode sensor, and the corresponding resonance frequencies were measured.

Figure 5C presents the computational simulation conducted to optimize the shape and size of the inductors. The external inductor must be large enough to facilitate long-range magnetic coupling. This is because increasing the inductor surface area allows the magnetic field to spread over a broader region, thereby enhancing the coupling coefficient with the reader coil (*66*). To prevent magnetic field cancellation, the inductor was positioned in a semicircular region around the stent. Consequently, the inductor was designed to have a vertically elongated shape proportional to the length of the stent. In addition to the scale of the inductor, the shape and size of the reader coil also influenced magnetic coupling. Therefore, the scale of the reader coil was considered a key analytical factor in the simulation. The simulation results showed that when the distance between the inductor and the reader coil was 100 mm, the S11 peak amplitude measured with a reader coil with the same size as that of the inductor was −0.2 dB. In contrast, when a reader coil twice the size of the inductor was used, the S11 peak decreased to −1.8 dB, indicating significantly stronger magnetic coupling with the larger reader coil (fig. S19). On the basis of these simulation results, a vertically elongated inductor and a reader coil twice the size of the inductor were selected for the wireless measurement system. The handheld external reader coil, designed for use in this LC-based wireless monitoring system, was optimized to enhance coupling efficiency (fig. S1).

Figure 5D shows a graph of the changes in the resonance frequency as the area covered by blood on the electrode varies from 0 to 100%. Initially, when only air was present in the electrode sensor (0%), the resonant frequency was 17.1 MHz. However, when the blood first covered the electrode at 12.5%, it drops to 13.2 MHz. As the area of the electrode sensor covered by blood increased, the resonant frequency decreased accordingly. Even with a slight increase in the covered area, the resonance frequency decreases considerably (at the megahertz range), demonstrating that sensitive detection is achievable wirelessly. A trend line plotted from the measured data revealed that the resonant frequency changes nonlinearly with the area covered by blood, which is consistent with the theoretical relationship in which the frequency is inversely proportional to the square root of the capacitance, thus confirming that the experimental results align with theoretical expectations.

As the stent is located deep within the body of the aorta, a considerable amount of biological tissue lies between the planar inductor and reader coil. To replicate these conditions, an S-parameter measurement was performed using a network analyzer with a 70-mm gap between the sensor inductor and reader coil, and the S11 parameters were measured in the frequency range of 1 to 20 MHz. The S11 data were processed by dividing the frequency axis into uniformly spaced bins. A representative value was selected within each bin to minimize noise and outliers, and the values were interpolated using a spline function to extract a smooth trend line (fig. S20). In addition, a phantom with the relative permittivity of muscle tissue (100) (*67*) was prepared and placed between the inductor and coil to simulate the transmission and reflection of electromagnetic waves through the biological tissue (fig. S1). Figure 5E shows the S11 measurements under these conditions, with the vessel and blood sequentially placed on the electrode sensor. When the vessel was placed, the resonance frequency was ~9 MHz, and when the blood was placed, it shifted to ~6 MHz, indicating a considerable resonance frequency shift. Notably, distinct peaks remained visible despite the substantial distance between the planar inductor and reader coil, implying that the sensor can reliably detect blood leakage, even when implanted in the body. Using this sensor-integrated stent, patients could rapidly monitor the entire process without visiting a hospital. In other words, patients with abdominal aortic aneurysms can substantially reduce the time and effort required to detect type 1a endoleaks.

## DISCUSSION

This study presented a capacitive electrode sensor for real-time monitoring of endoleaks to prevent aneurysmal recurrences. The thin and flexible capacitive sensor detected the capacitance difference between blood and blood vessels, showing a linear increase in capacitance even with residual blood volumes spanning thickness of 100 μm. In addition, it demonstrated high sensitivity and accuracy in measuring the heart rate and the location and velocity of blood flow within the blood pressure range of 80 to 120 mmHg. Experimental results indicated that the proposed 5-μm-thick sensor did not induce blood leakage. The film-type thermal adhesive provided strong bonding between the stent fabric and electrodes. The excellent stability of the proposed sensor was confirmed experimentally over 1000 compression/expansion cycles, maintaining consistent capacitance values and demonstrating stable performance even in the high-pressure environment of the aorta. Therefore, the proposed sensor demonstrated long-term stability of both the vessel and sensor under vascular, soft tissue, and dynamic conditions. Last, in addition to endoleak monitoring, future research may focus on the enhancement of sensor performance and exploration of its long-term in vivo applicability for monitoring various vascular diseases.

## MATERIALS AND METHODS

### Fabrication of the thin capacitance sensor

The process began with spin-casting of polymethyl methacrylate (PMMA; 950 PMMA A 2, Kayaku Advanced Materials), as a sacrificial layer, onto a glass substrate (MATSUNAMI, 1.2 to 1.5 mm), followed by curing at 180°C for 3 min. A layer of PI [poly(pyromellitic dianhydride-co-4,4′-oxydianiline), amic acid solution, Sigma-Aldrich] was spin-coated, baked at 150°C for 5 min, and fully cross-linked at 350°C under vacuum for 1 hour. An Au layer (thickness = 200 nm) was then deposited onto the PI-coated substrate using an electron beam evaporator. Photolithography (AZ 5214E, AZ Electronic Materials) patterning followed by wet metal etching processes created the Au electrode structures. Using the same PI recipe described above, a second layer of PI served as the top encapsulation layer for Au. After patterning the copper mask by photolithography and Cu wet etching, a reactive ion etcher (Samco Inc.) was used to remove the PI encapsulant. Immersion in boiling acetone (Samchun Pure Chemical) at 150°C for 1 hour removed the PMMA sacrificial layer.

### Fabrication of the thermal adhesive

Dulbecco’s phosphate-buffered saline (DPBS) (Sigma-Aldrich) and THF (Biograde, 99.8%, Thermo Fisher Scientific) were mixed at a 1:3 weight ratio. The Co-PA fabric (SINGER Iron-On Fusing web, Singer) was dissolved in a solvent mixture and heated at 130°C for 10 min. The resulting solution was spin-coated onto a glass substrate at 2000 rpm for 30 s and subsequently cooled to 20° to 25°C.

### Release and transfer of the thin capacitance sensor via adhesive

After removing the PMMA sacrificial layer, the electrode was separated from the substrate using water-soluble tape (AQUASOL, Aquasol Corporation). Heat was then applied to attach a heat adhesive layer to the underside of the electrode. The water-soluble tape was subsequently removed with deionized water, leaving the electrode adhered to the heat adhesive layer. Isopropyl alcohol (IPA) was applied to the stent fabric to temporarily fix the electrodes under surface tension. Last, heat was applied at 110°C using a flow gun to melt the heat adhesive, completing the bonding process.

### Adhesive properties of stent fabrics: Flexibility, adhesion strength, and penetration

PDMS (Sylgard 184, USA, with a base-to-curing agent ratio of 10:1), super glue (Mxbon 224902M), tissue liquid adhesive (Mastisol liquid adhesive), and a spray-on skin adhesive (Skinister Medical Adhesive Spray) were used as control groups for comparison. The adhesive developed in this study was temporarily bonded to the stent using IPA and thermally bonded via heat treatment. PDMS was applied to the fabric via spin coating (2000 rpm for 30 s) and then cured in an oven at 80°C for 2 hours. Super glue and medical adhesive were applied using spin coating (2000 rpm for 30 s) and cured at 30°C for 1 hour. The bending and adhesive strengths of the adhesives after curing were measured using a universal testing machine (AMETEK). To assess flexibility using the bending test, a compression force was applied at a speed of 50 mm/min to a two-layer stent fabric (20 mm by 60 mm) bonded to the adhesive. To evaluate the adhesive strength, two pieces of stent fabric (20 mm by 60 mm) were bonded using the adhesives, and a 180° peel test was conducted at a force of 5 N and speed of 60 mm/min. To assess the infiltration characteristics, adhesives were applied to the outer surface of the fabric, followed by the attachment of a PI film, and the inner surface was observed using SEM.

### Fabrication of gelatin gel for phantoms

A gel with electrical conductivities of 0.6 S/m (for blood) and 0.2 S/m (for blood vessels) was prepared according to the conductivity concentration formula. Gelatin powder (Herbnare) and NaCl (sodium chloride, ≥99%; Sigma-Aldrich) were dissolved in deionized water by heating on a hot plate at 90°C for 30 min. The solution was then cooled to 20° to 25°C in a 2-mm-deep glass mold, allowing the gel to form.

### Film thickness measurement and cyclic mechanical deformation testing of electrode

The thicknesses of the electrodes, including the metal layer and encapsulating PI, were measured using a surface profilometer (Alpha Step, Bruker). Compression and expansion stress testing of the electrodes was performed using a stent-crimping machine for more than 1000 cycles.

### Quantitative analysis of capacitance in vascular and blood phantoms

The capacitance of the coplanar capacitor was measured using a probe station over the frequency range of 0 to 200 kHz. Capacitance values were measured for equal volumes of blood, vessels, phantom material (vessel and blood, 10 mm by 15 mm by 2 mm), albumin (Officeahn), PBS (Hyunil Lab-Mate), and fat (Hanyang Sangsa). Electrode performance tests were conducted at a frequency of 100 kHz with the electrode covered in vascular gelatin to reproduce in vivo conditions. The residual blood height was controlled using molds with heights of 50, 100, and 200 μm for the blood phantom. The 10-μm-thick phantom layer, simulating vascular tissue, was adjusted by spinning at 200 rpm for 30 s using a spin coater, followed by curing and thickness measurement with an alpha step. To alter the blood flow speed, the electrode substrate was tilted at the angles of 0°, 45°, and 90°, and 1 ml of blood was injected into a 2-mm-wide PDMS (10:1) channel, resulting in blood flow speeds of 72, 172, and 860 μm/s, respectively.

### Thickness-dependent leakage analysis

A phantom simulating the elastic modulus of a blood vessel (0.5 MPa) was fabricated by mixing PDMS and Ecoflex at a ratio of 2:1. The mixture was poured into a 3 mm by 100 mm by 100 mm mold pretreated with a release agent and cured in a vacuum oven at 70°C for 2 hours. To create a temporary electrode with a total thickness of 1000 μm, PI tape (Kapton) with a thickness of 50 μm per layer was stacked into 20 layers. Temporary electrodes of varying thicknesses (sized 1.5 mm by 25 mm) were attached to the stent. The vascular phantom was shaped into a cylinder to enclose the stent with attached electrodes. To replicate the endoleak phenomenon, 0.1 ml of blood was injected into the upper area between the stent and vascular phantom using a syringe. The blood pressure environment was simulated by inserting a vinyl tube inside the stent and applying an internal pressure of 80 to 120 mmHg at a rate of 60 to 100 bpm, using an air pump.

### Long-term stability assessment

The stent with electrodes was fully immersed in a PBS solution at 37°C and observed every 3 days for up to 1 month. Before measuring capacitance, the stent was removed from the PBS solution and dried at 20° to 25°C for ~30 min. The measurement tips of the probe station were connected to the contact pads of a short electrode pair with a length of 13.5 mm. After obtaining the air data, a blood phantom (10 mm by 5 mm) was placed on the electrode pair to obtain blood data.

### Toxicity assessment of electrode sensor and adhesive

Endothelial growth medium-2 (EGM-2, LONZA) was used for HUVECs cultures under 5% CO_2_ conditions at 37°C. Conditioned media containing potential extracts of PI and Co-PA films were prepared according to a standardized protocol (ISO 10993-12). Specifically, the film with an area of 600 mm^2^ was placed onto a 24-well plate, subjected to incubation for 24 hours at a temperature of 37°C in 1 ml of culture media, and the conditioned media were then collected. The cytotoxicity evaluation of the engineered films involved the cultivation of HUVECs in conditioned media for 1 day. Normal EGM-2 cells were used as the controls. For live cell staining, the cells were treated with DPBS containing calcein-AM (1:1000) for 10 min (live/dead viability kit, Invitrogen), and the samples were subsequently observed under a fluorescent microscope (Eclipse Ti2, Nikon; Tokyo, Japan). For the MTT assay, we incubated cells with an MTT solution for 1 hour at 37°C, and the resulting MTT formazans were extracted using dimethyl sulfoxide (Sigma-Aldrich). The absorbance was measured at 550 nm using a microplate reader (Varioskan LUX, Thermo Fisher Scientific; Waltham, MA, USA).

### Ex vivo experimental setup

A stent integrated with the sensor was inserted into the porcine aorta using a catheter (Medtronic). To reproduce physiological blood pressure conditions, a vinyl tube was placed inside the stent, and an air pump was used to apply pressures in the range of 80 to 120 mmHg at a heart rate range of 80 to 90 bpm. A syringe (Jibengao, 1 ml) was positioned between the vessel and the stent, and 0.5 ml each of blood, protein, PBS, and lipids was sequentially injected at a temperature of 37°C in a controlled environment.

### HFSS simulation to optimize the size of planar inductor and reader coil

The commercial software ANSYS HFSS (High-Frequency Structure Simulator) was used to perform three-dimensional electromagnetic finite element analysis of the receiver (Rx) and transmitter (Tx) in a wireless sensing system. This analysis determined the electromagnetic coupling, scattering coefficient (S11), and resonant frequency. The Rx coil was modeled as a planar, multiturn (*N* = 3) rectangular inductive coil with dimensions of *L* = 100 mm and *W* = 40 mm, while the Tx coil was modeled for two representative designs: (i) as a single-turn rectangular wire antenna with dimensions of *L* = 110 mm and *W* = 50 and 20 mm and with dimensions of *L* = 200 mm and *W* = 120 mm. The design was optimized to maximize the area coverage between the Rx and Tx wireless systems and achieve a sensing and detection distance of up to 100 mm. The Rx antenna has an external capacitor (*C* = 117 pF) to resonate at a frequency *f* between 13.3 and 13.4 MHz. An adaptive mesh (tetrahedron elements) and a spherical radiation boundary (radius, 1000 mm) were adopted to ensure computational accuracy and convergence. The nonlinear relationship between the S11 parameter (at resonance) and the separation distance was calculated at over 60 to 100 mm. The material used in the simulation for the Rx and Tx is copper with an electrical conductivity of σ_Cu_ = 5.96 × 10^7^ S/m, and all the simulations were performed on air.

### Setup for wireless monitoring and fabrication of phantom

The inductor, connected to long electrode pairs and an anisotropic conductive film to form an LC resonance circuit, was fabricated by etching a cross-sectional flexible printed circuit board with copper (18 μm) and PI (25 μm) using a laser cutter (LPKF PL U4). The inductor dimensions were 30 mm by 100 mm, with 13 turns, and both spacing and width were 400 μm. A rectangular copper coil (2.6 mm; diameter: 60 mm by 200 mm) was connected to the network analyzer via a Sub-Miniature Version A (SMA) connector (SubMiniature version A connector). The S11 parameters were measured using a network analyzer in the frequency range of 1 to 20 MHz at a sampling rate of 30 kHz. To process the raw S11 data and extract a smooth trend line, the frequency axis was divided into uniformly spaced bins. Within each bin, a representative value was selected to mitigate the effects of noise and outliers. These values were subsequently interpolated using a spline function, preserving the characteristic dip in the S11 response while producing a smooth overall curve. A dielectric muscle phantom with a thickness of 70 mm was placed between the sensor inductor and coil. Phantom was prepared by pouring 20% gelatin into a mold of dimensions 100 mm by 100 mm by 15 mm and allowing it to solidify in a refrigerator at 4°C for 24 hours. A circular hole with a diameter of 30 mm was created on the side of the solidified gelatine, which was then stacked at a height of 150 mm.

## Supplementary Materials

### This PDF file includes:

Supplementary Text Figs. S1 to S20 References
