# Supplementary material for: A wireless, implantable sensor for continuous monitoring of blood leakage after endovascular aneurysm repair
Source: Sci Adv. 2025 Oct 1;11(40):eady6148. doi: 10.1126/sciadv.ady6148 (PMC12487876; doi:10.1126/sciadv.ady6148)
Supplement: Supplementary file 1 — Supplementary Text Figs. S1 to S20 References [file sciadv.ady6148_sm.pdf]

Science Advances
AAAS

Supplementary Materials for

A wireless, implantable sensor for continuous monitoring of blood leakage after endovascular aneurysm repair

Sun Young Park *et al.*

Corresponding author: Yei Hwan Jung, yjung@hanyang.ac.kr; Young-Hyo Lim, mdoim@hanyang.ac.kr

Sci. Adv. **11**, eady6148 (2025)
DOI: 10.1126/sciadv.ady6148

This PDF file includes:

Supplementary Text
Figs. S1 to S20
References

Supplementary Text

1. Computational Fluid Dynamics Settings

1.1. Basic Information and Geometry Description for Simulated Cases

The dimensions for the two-dimensional aorta and stent mesh design were based on the study by (*68*) using Ansys Fluent (2023R2) and Ansys Meshing (2023R2). A total of six different gap sizes were selected for CFD simulation, including 5μm, which corresponds to the actual gap size measured by endoleak detection sensors. Simulations were conducted for six cases with mesh gap sizes of 5μm, 10μm, 50μm, 100μm, 500μm, and 1000μm. Except for the gap size variations, all simulation conditions were kept consistent across cases.

1.2. Simulation Settings: Flow Characteristics and Boundary Conditions

The flow type was set as laminar, and the fluid density was assigned a value of 1060 kg/m³, representing the density of human blood. Three types of boundaries were defined:

(i) Stent and Aorta Walls: No-slip boundary condition.

(ii) Inlet: Velocity inlet with a 120 bpm pulsatile blood flow. A user-defined function (UDF) was employed to simulate this inlet condition using the following equation:

$$v_{inlet}(t) = \begin{cases} 0.5\sin\big(4\pi(t + 0.0160236)\big) & 0.5n < t \le 0.5n + 0.218 \\ 0.1 & 0.5n + 0.218 < t \le 0.5(n+1) \end{cases} \quad (1)$$

Where n is an natural number which indicates the number of pulsating flows.

(iii) Outlet: The outlet was set as a pressure outlet with an outlet pressure of 13,332 Pa.

1.3. Carreau Viscosity Model

The Carreau viscosity model was employed to simulate laminar blood flow, prescribing non-Newtonian viscosity characteristics of the blood flow. This approach satisfies the conditions for internal flow in the stent and between the stent and aorta, which were confirmed as laminar in post-validation tests (*36*). The blood viscosity is described by the following equation:

$$\mu_{eff}(\dot{r}) = \mu_{inf} + (\mu_0 - \mu_{inf})(1 + (\lambda\dot{r})^2)^{\frac{n-1}{2}} \quad (2)$$

In equation 2, μ_{inf} is the viscosity at infinite shear rate, μ_0 is the viscosity at zero shear rate, λ is the characteristic time and n is the power index. μ_{inf}, μ_0, λ, and n are assumed constant. In the simulation used constants values were, $\mu_{inf} = 0.0035\ (kg/m \cdot s)$, $\mu_0 = 0.056\ (kg/m \cdot s)$, $\lambda = 3.313(s)$, and $n = 0.3568$. The selection process for these constants was based on (*69*).

2. Analytical approach

An analytical approach was considered to solve the relationship between the blood flow velocity, V_2, and gap size created by aorta and stent system. This model approximates blood flow through the aorta and stent system as an internal-pipe laminar flow. The modified Darcy–Weisbach equation, which accounts for the bifurcating blood flow across various gap thicknesses, is expressed as

$$\left[\frac{f_1 L_1}{2g d_i} + \frac{f_1' L_1'}{2g d_i'} \cdot \left\{\frac{\frac{\pi}{4} d_i^2}{2 \cdot \frac{\pi}{4} d_i'^2}\right\}^2\right] \cdot V_1^2 = \left[\frac{f_2 L_2}{2g D_h} + \frac{f_2' L_2'}{2g D_h'} \cdot \left\{\frac{\frac{\pi}{4}(d_o^2 - d_w^2)}{2 \cdot \frac{\pi}{4}(d_o'^2 - d_w'^2)}\right\}^2\right] \cdot V_2^2 \quad (3)$$

where f_1, V_1, L_1 denote the Darcy friction factor, flow velocity, and length of the stent, respectively. Similarly, f_2, V_2, L_2 pertain to the flow through the gap between the stent and arterial wall. D_h represents the hydraulic diameter of the gap, and g is the gravitational constant. The symbols d_i, d_o, d_w represent the inner, outer, and inner diameters of the stent and aorta, respectively (Fig. S10). Superscript primes indicate parameters after bifurcation.

The analytical model assumes that the head loss of flow through the stent and the flow through the gap between the aorta and stent are identical. The model also assumes that viscosity of blood can be expressed by the Carreau viscosity model. Equation (3) was solved iteratively, with the terms multiplied by V_1^2 and V_2^2 treated as functions of V_2. During the calculations, $V_{2,n}$ was computed based on $V_{2,n-1}$ as expressed by Equation (4):

$$V_{2,n} = \sqrt{\frac{C_1(V_{2,n-1})}{C_2(V_{2,n-1})}} V_{1,n-1}(V_{2,n-1}) \quad (4)$$

where C_1 and C_2 are the terms multiplied by V_1^2 and V_2^2 in equation (3). The detailed analysis process continues below.

2.1. Definition of head loss of internal pipe flow

Head loss of pipe flow defined as below equation (*50*)

$$h_f = f \cdot \frac{LV^2}{2gd} \quad (5)$$

In equation (5), f is darcy friction factor, L is pipe length, V is flow velocity, and d is pipe diameter.

For laminar flow in a circular pipe, the Darcy friction factor f can be expressed explicitly as:

$$f = \frac{64}{Re_D} \quad (6)$$

For flow through a concentric annulus, the friction factor f is modified as:

$$f = \frac{64\zeta}{Re_D} \quad (7)$$

Where ζ is given by

$$\zeta = \frac{(a-b)^2(a^2-b^2)}{a^4 - b^4 - (a^2-b^2)^2/\ln\left(\frac{a}{b}\right)} \quad (8)$$

Here, a is outer radius, and b is inner radius of concentric annulus. Re_D is Reynolds number, using diameter as characteristic length. Reynolds number is dimensionless number representing the ratio between inertial force and viscous force. Reynolds number is defined as

$$Re_D = \frac{\rho VD}{\mu} \quad (9)$$

While ρ is density, μ is viscosity of fluid. For internal flow, when Re_D is smaller than 2000, the flow is considered laminar.

2.2. Hydraulic diameter for concentric annulus

According to (*70*), hydraulic diameter for concentric annulus is

$$D_h = 2\,(a-b) \quad (10)$$

2.3. Head loss equation

Based on the geometry of the system, blood flow from the inlet bifurcates into two branches. One branch flows through the stent and the other branch flows through the gap, which is considered as a concentric annulus. Since both flow paths rejoin after bifurcation, they must experience the same head loss.

2.3.1. Relationship between V_1 and V_2

If V_0 is flow velocity at the inlet, and Q_0 is volume flow rate at the inlet, then,

$$Q_0 = \frac{\pi}{4} d_w^2 V_0 = \frac{\pi}{4} d_i^2 \cdot V_1 + \frac{\pi}{4}(d_w^2 - d_o^2) \cdot V_2 \quad (11)$$

2.3.2. Head loss equation for flow through the stent and the gap

The head loss equation for flow through the stent and the flow through the gap is

$$\frac{f_1 L_1}{2gd_i}V_1^2 + \frac{f_1' L_1'}{2gd_i'}V_1'^2 = \frac{f_2 L_2}{2gD_h}V_2^2 + \frac{f_2' L_2'}{2gD_h'}V_2'^2 \qquad (12)$$

If Q_1 and Q_1' represent the volume flow rates through the stent before and after bifurcation, and Q_2 and Q_2' represent those for the gap, then

$$Q_1 = \frac{\pi}{4}d_i^2 \cdot V_1 \qquad (13)$$

$$Q_1' = \frac{\pi}{4}d_i'^2 \cdot V_1' \qquad (14)$$

$$Q_2 = \frac{\pi}{4}(d_w - d_o) \cdot V_2 \qquad (15)$$

$$Q_2' = \frac{\pi}{4}(d_w' - d_o'^2) \cdot V_2' \qquad (16)$$

Based on geometry, the equations below are true

$$Q_1 = 2Q_1' \quad (17) \text{ and } Q_2 = 2Q_2' \quad (18)$$

Finally, V_1' and V_2' can be expressed in terms of V_1 and V_2

$$V_1' = \frac{d_i^2}{2 \cdot d_i'^2} V_1 \quad (19) \text{ and } V_2' = \frac{Q_2}{Q_2'} \cdot V_2 = \frac{(d_w^2 - d_o)}{2(d_o'^2 - d_o'^2)} V_2 \quad (20)$$

Thus, the darcy friction factors f_1, f_1', f_2 and f_2' can be expressed using only V_1 and V_2. By substituting the corresponding variable values into the equation (6) and (7),

$$f_1 = \frac{64}{Re_D} = \frac{64\mu}{\rho V_1 d_i} \qquad (21)$$

$$f_1' = \frac{64}{Re_D} = \frac{64\mu}{\rho V_1' d_i'} = \frac{128\mu \cdot d_i'}{\rho \cdot d_i^2 \cdot V_1 \cdot d_i'} \qquad (22)$$

$$f_2 = \frac{64\zeta}{Re_D} = \frac{64\mu \cdot \zeta}{\rho V_2 D_h} \qquad (23)$$

$$f_2' = \frac{64\zeta}{Re_D} = \frac{64\mu \cdot \zeta}{\rho V_2' D_h'} = \frac{128\mu \cdot (d_o'^2 - d_o'^2)}{\rho \cdot (d_w^2 - d_o) \cdot V_2 \cdot D_h'} \qquad (24)$$

Where viscosity μ follows equation (2).

The hydraulic diameters of the gap before and after bifurcation D_h and D_h' are given by

$$D_h = 2\left(\frac{d_w}{2} - \frac{d_o}{2}\right) \quad (25) \quad \text{and} \quad D_h' = 2\left(\frac{d_w'}{2} - \frac{d_o'}{2}\right) \quad (26)$$

Therefore, head loss equation includes only unknown variables V_1 and V_2.

$$\left[\frac{f_1 L_1}{2gd_i} + \frac{f_1' L_1'}{2gd_i'} \cdot \left\{\frac{\frac{\pi}{4}d_i^2}{2 \cdot \frac{\pi}{4}d_i'^2}\right\}^2\right] \cdot V_1^2 = \left[\frac{f_2 L_2}{2gD_h} + \frac{f_2' L_2'}{2gD_h'} \cdot \left\{\frac{\frac{\pi}{4}(d_w^2 - d_o)}{2 \cdot \frac{\pi}{4}(d_w'^2 - d_o'^2)}\right\}^2\right] \cdot V_2^2 \qquad (27)$$

2.4. Implicit formula to calculate V_2

From equation (9), V_1 can be expressed in terms of V_2

$$V_1 = \frac{4Q_0}{\pi d_i} - \frac{d_w^2 - d_o^2}{d_i^2} V_2 \qquad (28)$$

Thus, V_1 can be considered as a function of V_2.

If the terms multiplied to V_1^2 and V_2^2 in the equation (27) are denoted as C_1 and C_2, then, from equation (27),

$$V_2 = \sqrt{\frac{C_1}{C_2}} V_1 \qquad (29)$$

Although C_1 is not the function of V_2, it can be computed using V_2 since V_1 is the function of V_2 and all other parameters are known. Therefore, using equation (29) and an iteration method, V_2 can be calculated.

2.5. Iterative method

The iterative calculation was performed using Jupiter Notebook (v 7.0.8) an open-source Python software. The iteration was carried out using Equation (29), rewritten in the form of Equation (30):

$$V_{2,n} = \sqrt{\frac{C_1(V_{2,n-1})}{C_2(V_{2,n-1})}} V_{1,n-1}(V_{2,n-1}) \quad (30)$$

In the in-house python code, $C_1(V_{2,n-1})$, $C_2(V_{2,n-1})$ and $V_{1,n-1}(V_{2,n-1})$ were treated as functions of $V_{2,n-1}$. The iteration continued until the error between $V_{2,n}$ and $V_{2,n-1}$ became less than 0.01%

2.6. Simulation settings for modeling the non-Newtonian behavior of blood

The simulation settings are as follows:

The flow is set to laminar

The viscosity is modeled using the Carreau model

$$\mu = \mu_{inf} + (\mu_0 - \mu_{inf})(1 + (\lambda\dot{\gamma})^2)^{\frac{n-1}{2}}$$

$$\begin{cases} \lambda = 3.313(s \\ n = 0.3568 \\ \mu_0 = 0.056\ (kg/m \cdot s) \\ \mu_{inf} = 0.0035(kg/m \cdot s) \end{cases}$$

Blood, $\rho = 1060 kg/m^3$

PBS, $\rho = 1084 kg/m^3$, $\mu = 1.27 \times 10^{-3} kg/m \cdot s$

Albumin, $\rho = 1017 kg/m^3$, $\mu = 1.86 \times 10^{-3} kg/m \cdot s$ (*71*)

Boundary condition:

Wall: Non-slip condition, where the fluid velocity at the wall is zero.

Inlet: A blood flow rate of 120 beats per minute (bpm),

$$v_{inlet}(t) = \begin{cases} 0.5 \sin(4\pi(t + 0.0160236)) & 0.5n < t \leq 0.5n + 0.218 \\ 0.1 & 0.5n + 0.218 < t \leq 0.5(n+1) \end{cases}$$

Outlet: Pressure outlet with a pressure of 13332 Pa.

Fig. S1.

LC-based wireless monitoring system. (a) Handheld external reader coil used in an LC-based wireless monitoring system. (b) Experimental setup for LC-based wireless monitoring using a muscle-mimicking phantom and an inductive reader coil. (c) Radiopaque marker integrated into the stent graft, serving as a fixed reference point for imaging-based localization after implantation.

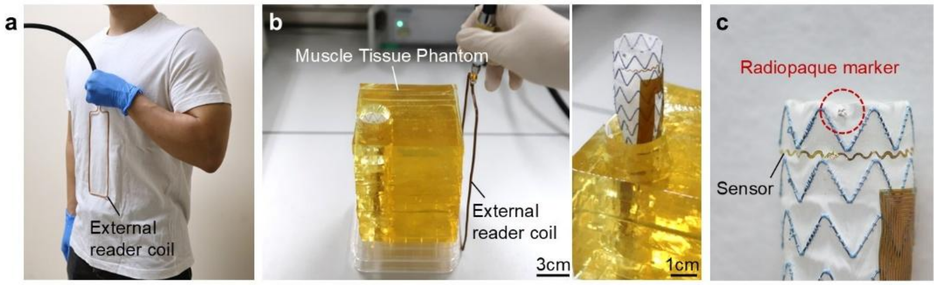

Fig. S2.

Thickness data of the capacitive sensor. (a) Graph showing the overall sensor thickness measured using an alpha step profiler. (b) Photograph of the sensor placed between stent struts with injected blood; the sensor is thinner than the stent, preventing blood leakage.

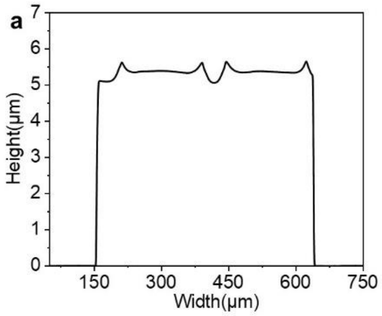

Fig. S3.

Graph showing the capacitance of the vessel and blood depending on the serpentine degree of the sensor. The angles are 120°, 180°, and 240°.

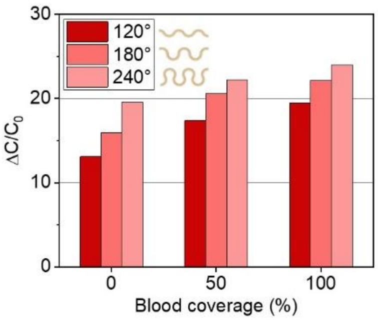

Fig. S4.

Capacitance of biological substances measured across the 0-200 kHz frequency range. The measurements included air, blood, vessel, mimicking phantom (blood, vessel), fat, and albumin.

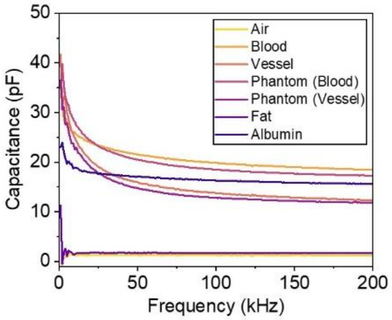

Fig. S5.

Photograph of the vessel and blood gelatin phantom used in the experiment. Scale bars, 1 cm.

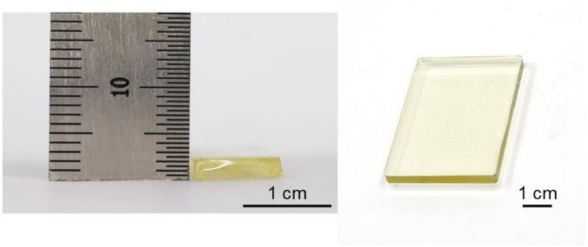

Fig. S6.

Capacitance-based blood leakage detection in the presence of thin fibrin mimics.
Capacitance changes induced by blood were measured at 100 kHz after applying thin fibrin-mimicking layers (10 μm and 100 μm) to the phantom surface.

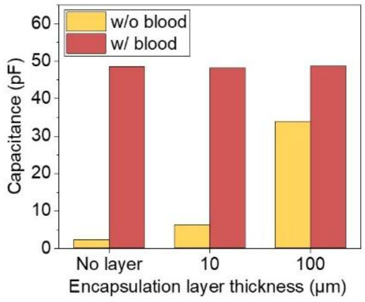

Fig. S7.

Graph showing the measured capacitance in three conditions: (1) blood flow inside the stent graft, (2) vessel in contact with the sensor attached to the outer surface of the stent graft, and (3) blood in contact with the sensor on the outer surface.

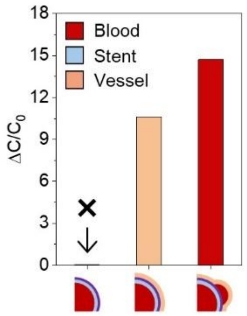

Fig. S8.

Capacitance response of the sensor under single-site and multi-site blood leakage conditions. The capacitance of each electrode was recorded under no leakage, single-site with two locations, and multi-site conditions.

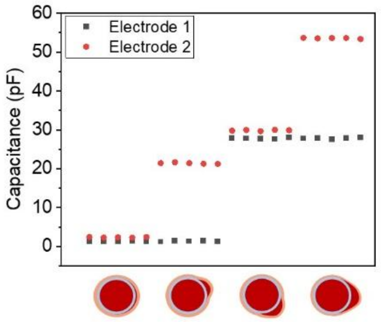

Fig. S9.

Capacitance measured during compression and expansion of the stent graft with the sensor attached to the stent. The stent crimping equipment was used.

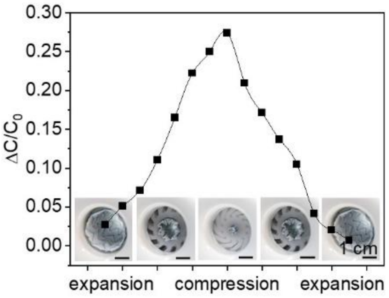

Fig. S10.

Capacitance measured during the compression and expansion of the stent graft with the attached sensor. The measurements were taken using a stent-crimping machine. This process was repeated over 100 cycles.

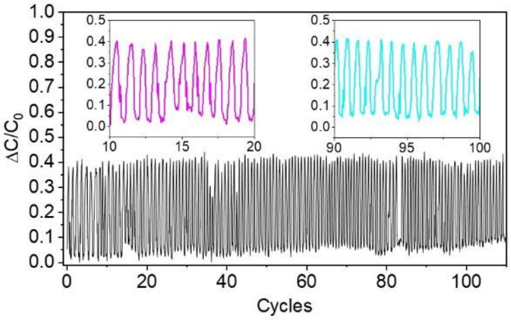

Fig. S11.

Schematic of the ex vivo experimental setup. (a) Schematic of the endoleak reproduced with a syringe and blood. (b) Actual stent with a scale bar of 1 cm. (c) Vessel and inner surface used in the experiment with scale bars of 1 cm and 5 mm.

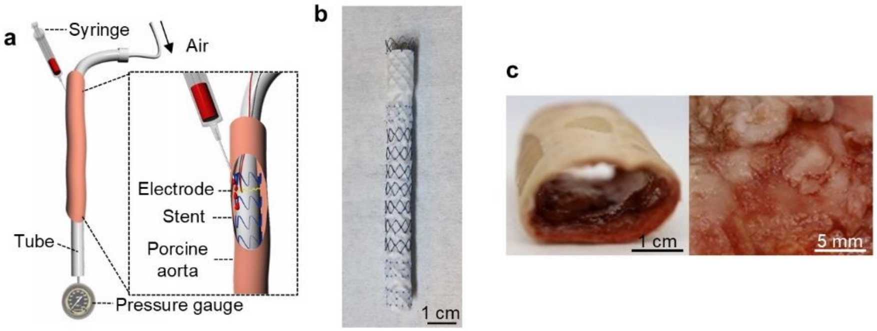

Fig. S12.

Simulation of signal fluctuations induced by the non-Newtonian behavior of blood. Blood viscosity was modeled using the Carreau model to account for shear rate-dependent changes, reflecting the heterogeneous composition of blood, including red blood cells.

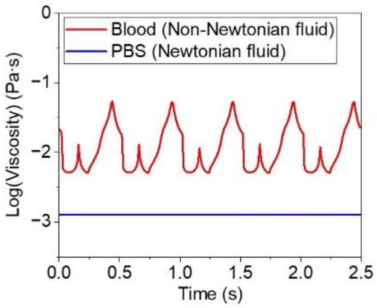

Fig. S13.

Capacitance of vascular tissue and blood leakage in a porcine vascular system was measured ex vivo. The measurements were taken across physiological (80–120 mmHg) and hypertensive (100–180 mmHg) pressure ranges.

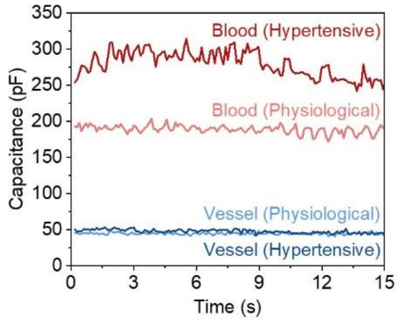

Fig. S14.

Characterization of electrode thickness, peel strength, and images bonded to stents after PBS immersion. (a) Thickness measurements measured using an alpha-step profiler after immersion in PBS for 0, 1, 7, and 14 days. (b) Peel strength of electrodes bonded to stents after PBS immersion for 0, 1, and 7 days. (c) Images of electrodes bonded to stents after immersion in PBS at 95 °C for 0, 7, 14, and 21 days.

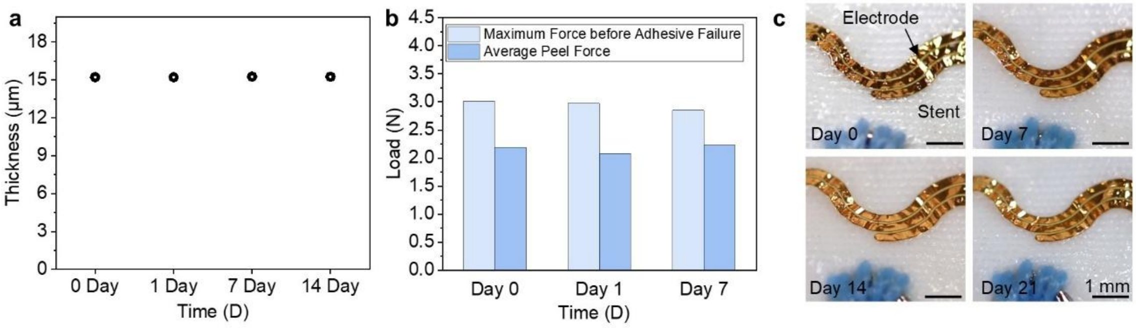

Fig. S15.

SEM images of stent fabric and sensor-fabric interface. (a) Cross-sectional SEM image of the stent fabric treated with the proposed adhesive. (b) SEM image of the sensor–fabric interface bonded using the proposed adhesive. (c) SEM image of the sensor–fabric interface after 1,000 cycles of substantial compression and expansion using a clamping device.

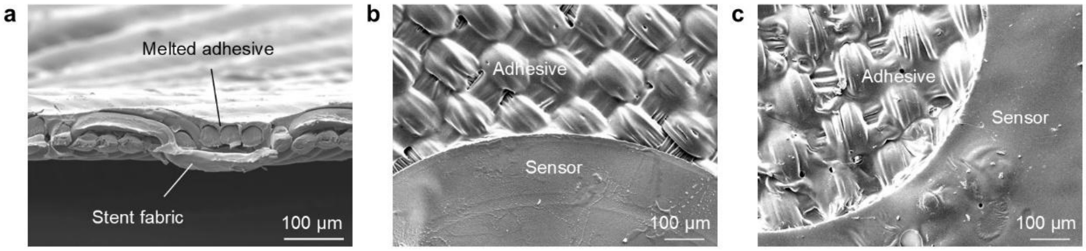

Fig. S16.

Photographs of blood droplets on stent graft fabrics treated with different adhesives. After applying thermal adhesive, PDMS, and instant adhesive to the stent graft fabric, sheep blood was applied to the surface. Scale bars, 1 mm.

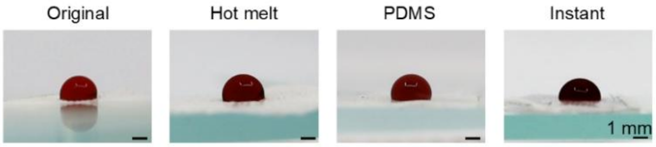

Fig. S17.

Simulation of blood flow through the aorta and stent. (a) Schematic illustration of the analytical model describing blood flow through the aorta and stent system. (b) Mesh configuration of the computational domain.

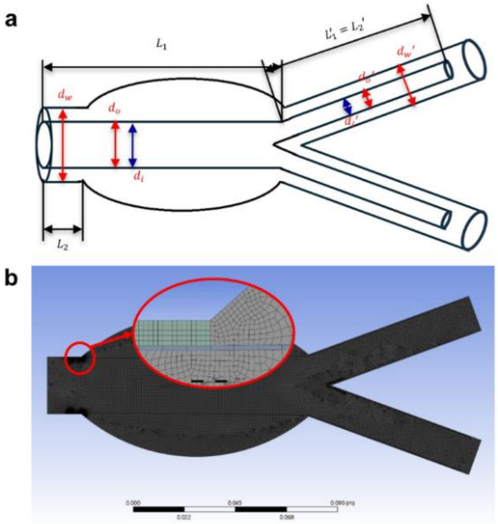

Fig. S18.

S11 response of the LC wireless system measured. Measured on a styrofoam block (air-like reference) and on a stent.

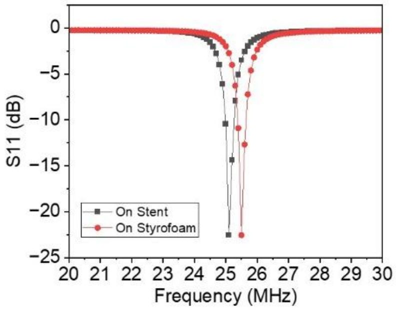

Fig. S19.

HFSS simulation results. (a) Reader coil with the same diameter as the implanted inductor. (b) Reader coil with twice the diameter of the implanted inductor. (c) Comparison of resonance peak magnitudes between the two configurations.

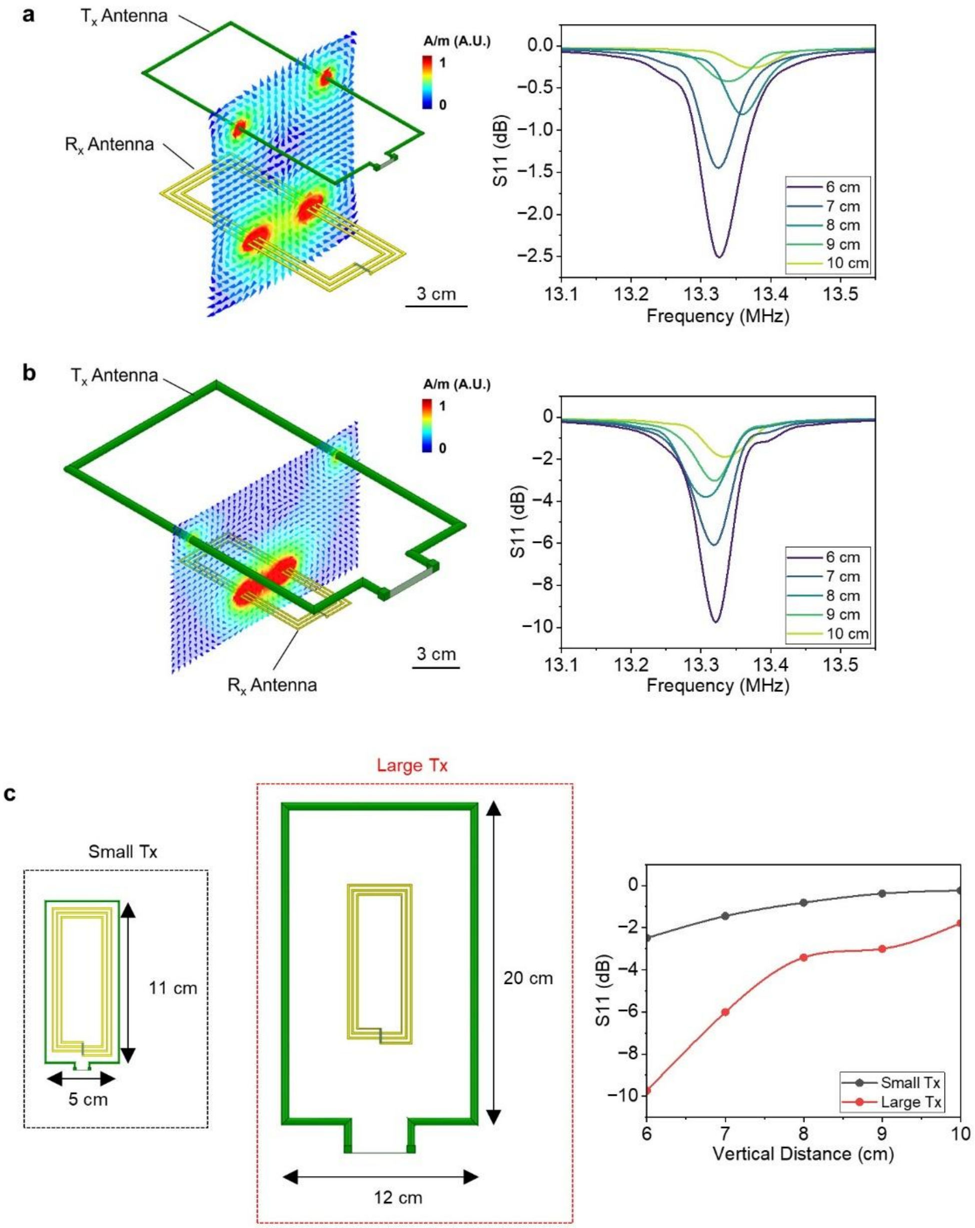

Fig. S20.

S11 data processed by dividing the frequency axis into uniformly spaced bins. A representative value was selected within each bin to minimize noise and outliers, and the values were interpolated using a spline function to extract a smooth trend line.

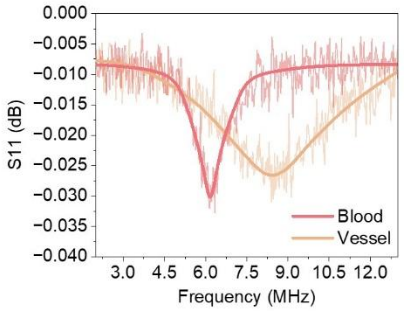

REFERENCES AND NOTES

1. J. Parodi, J. Palmaz, H. Barone, Transfemoral intraluminal graft implantation for abdominal aortic aneurysms. *Ann. Vasc. Surg.* **5**, 491–499 (1991).

2. A. Odero, V. Arici, A. Bozzani, Endovascular repair of abdominal aortic aneurysm. *N. Engl. J. Med.* **363**, 1479–1482 (2010).

3. A. Sharma, P. Sethi, K. Gupta, Endovascular abdominal aortic aneurysm repair. *Interv. Cardiol. Clin.* **9**, 153–168 (2020).

4. R. M. Greenhalgh, L. C. Brown, G. P. S. Kwong, J. T. Powell, S. G. Thompson, EVAR trial participants, Comparison of endovascular aneurysm repair with open repair in patients with abdominal aortic aneurysm (EVAR trial 1), 30-day operative mortality results: Randomised controlled trial. *Lancet* **364**, 843–848 (2004).

5. F. A. Lederle, J. A. Freischlag, T. C. Kyriakides, F. T. Padberg Jr., J. S. Matsumura, T. R. Kohler, P. H. Lin, J. M. Jean-Claude, D. F. Cikrit, K. M. Swanson, P. N. Peduzzi, Open Versus Endovascular Repair (OVER) Veterans Affairs Cooperative Study Group, Outcomes following endovascular vs open repair of abdominal aortic aneurysm: A randomized trial. *JAMA* **302**, 1535–1542 (2009).

6. M. Prinssen, E. L. G. Verhoeven, J. Buth, P. W. M. Cuypers, M. R. H. M. van Sambeek, R. Balm, E. Buskens, D. E. Grobbee, J. D. Blankensteijn, A randomized trial comparing conventional and endovascular repair of abdominal aortic aneurysms. *N. Engl. J. Med.* **351**, 1607–1618 (2004).

7. G. H. White, W. Yu, J. May, To the editors:"Endoleak"—A proposed new terminology to describe incomplete aneurysm exclusion by an endoluminal graft. *J. Endovasc. Ther.* **3**, 124–125 (1996).

8. G. H. White, W. Yu, J. May, X. Chaufour, M. S. Stephen, Endoleak as a complication of endoluminal grafting of abdominal aortic aneurysms: Classification, incidence, diagnosis, and management. *J. Endovasc. Surg.* **4**, 152–168 (1997).

9. M. Boufi, F. Aouini, C. Guivier-Curien, B. Dona, A. D. Loundou, V. Deplano, Y. S. Alimi, Examination of factors in type I endoleak development after thoracic endovascular repair. *J. Vasc. Surg.* **61**, 317–323 (2015).

10. I. Mohan, R. Laheij, P. Harris, Risk factors for endoleak and the evidence for stent-graft oversizing in patients undergoing endovascular aneurysm repair. *Eur. J. Vasc. Endovasc. Surg.* **21**, 344–349 (2001).

11. S. M. Sampaio, J. M. Panneton, G. I. Mozes, J. C. Andrews, T. C. Bower, M. Karla, A. A. Noel, K. J. Cherry, T. Sullivan, P. Gloviczki, Proximal type I endoleak after endovascular abdominal aortic aneurysm repair: Predictive factors. *Ann. Vasc. Surg.* **18**, 621–628 (2004).

12. G. H. White, J. May, R. C. Waugh, W. Yu, Type I and type II endoleaks: A more useful classification for reporting results of endoluminal AAA repair. *J. Endovasc. Ther.* **5**, 189–191 (1998).

13. J. C. Parodi, R. Berguer, L. M. Ferreira, R. La Mura, M. L. Schermerhorn, Intra-aneurysmal pressure after incomplete endovascular exclusion. *J. Vasc. Surg.* **34**, 909–914 (2001).

14. T.-W. Tan, M. Eslami, D. Rybin, G. Doros, W. W. Zhang, A. Farber, Outcomes of patients with type I endoleak at completion of endovascular abdominal aneurysm repair. *J. Vasc. Surg.* **63**, 1420–1427 (2016).

15. M. Jones, P. Faris, R. Moore, Mortality and risk factors for ruptured abdominal aortic aneurysm after repair endovascular (rARE). *J. Vasc. Surg. Cases Innov. Tech.* **9**, 101165 (2023).

16. P. Cao, P. De Rango, F. Verzini, G. Parlani, Endoleak after endovascular aortic repair: Classification, diagnosis and management following endovascular thoracic and abdominal aortic repair. *J. Card. Surg.* **51**, 53–69 (2010).

17. M. D. Hiatt, G. D. Rubin, Surveillance for endoleaks: How to detect all of them. *Semin. Vasc. Surg.* **17**, 268–278 (2004).

18. S. W. Stavropoulos, R. A. Baum, Imaging modalities for the detection and management of endoleaks. *Semin. Vasc. Surg.* **17**, 154–160 (2004).

19. S. W. Stavropoulos, S. R. Charagundla, Imaging techniques for detection and management of endoleaks after endovascular aortic aneurysm repair. *Radiology* **243**, 641–655 (2007).

20. J. H. Ahn, J. Y. Kim, Y. S. Jeon, S. G. Cho, J. K. Park, K. J. Lee, K. C. Hong, Successful treatment of type I endoleak of common iliac artery with balloon expandable stent (Palmaz XL stent) during endovascular aneurysm repair. *J. Korean Surg. Soc.* **82**, 59–62 (2012).

21. A. C. Picel, N. Kansal, Essentials of endovascular abdominal aortic aneurysm repair imaging: Postprocedure surveillance and complications. *Am. J. Roentgenol.* **203**, W358–W372 (2014).

22. E. L. Chaikof, R. L. Dalman, M. K. Eskandari, B. M. Jackson, W. A. Lee, M. A. Mansour, T. M. Mastracci, M. Mell, M. H. Murad, L. L. Nguyen, G. S. Oderich, M. S. Patel, M. L. Schermerhorn, B. W. Starnes, The Society for Vascular Surgery practice guidelines on the care of patients with an abdominal aortic aneurysm. *J. Vasc. Surg.* **67**, 2–77.e2 (2018).

23. E. A. Andrasksa, A. R. Phillips, K. M. Reitz, S. Asaadi, Y. Dai, E. Tzeng, M. Makaroun, N. L. Liang, Longer follow-up intervals after EVAR is safe and appropriate after marked aneurysm sac regression. *J. Vasc. Surg.* **74**, e88–e89 (2021).

24. A. Schanzer, R. K. Greenberg, N. Hevelone, W. P. Robinson, M. H. Eslami, R. J. Goldberg, L. Messina, Predictors of abdominal aortic aneurysm sac enlargement after endovascular repair. *Circulation* **123**, 2848–2855 (2011).

25. Writing Committee Members, E. M. Isselbacher, O. Preventza, J. H. Black III, J. G. Augoustides, A. W. Beck, M. A. Bolen, A. C. Braverman, B. E. Bray, M. M. Brown-Zimmerman, E. P. Chen, T. J. Collins, Abe De Anda Jr, C. L. Fanola, L. N. Girardi, C. W. Hicks, D. S. Hui, W. S. Jones, V. Kalahasti, K. M. Kim, D. M. Milewicz, G. S. Oderich, L. Ogbechie, S. B. Promes, E. G. Ross, M. L. Schermerhorn, S. S. Times, E. E. Tseng, G. J. Wang, Y. J. Woo, 2022 ACC/AHA guideline for the diagnosis and management of aortic disease: A report of the

American Heart Association/American College of Cardiology Joint Committee on Clinical Practice Guidelines. *J. Am. Coll. Cardiol.* **80**, e223–e393 (2022).

26. T. Ide, K. Masada, T. Kuratani, R. Sakaniwa, K. Shimamura, K. Kin, Y. Watanabe, R. Matsumoto, Y. Sawa, Risk analysis of aneurysm sac enlargement caused by type II endoleak after endovascular aortic repair. *Ann. Vasc. Surg.* **77**, 208–216 (2021).

27. S. A. Dingemans, F. H. Jonker, F. L. Moll, J. A. van Herwaarden, Aneurysm sac enlargement after endovascular abdominal aortic aneurysm repair. *Ann. Vasc. Surg.* **31**, 229–238 (2016).

28. L. K. Baxter, *Capacitive Sensors: Design and Applications* (Wiley, 1996).

29. W. C. Heerens, Application of capacitance techniques in sensor design. *J. Phys. E Sci. Instrum.* **19**, 897–906 (1986).

30. R. Nopper, R. Niekrawietz, L. Reindl, Wireless readout of passive LC sensors. *IEEE Trans. Instrum. Meas.* **59**, 2450–2457 (2010).

31. E. Tzortzis, R. J. Hinchliffe, B. R. Hopkinson, Adjunctive procedures for the treatment of proximal type I endoleak: The role of peri-aortic ligatures and Palmaz stenting. *J. Endovasc. Ther.* **10**, 233–239 (2003).

32. M. H. Seelig, W. A. Oldenburg, A. G. Hakaim, J. W. Hallett, A. Chowla, J. C. Andrews, K. J. Cherry, Endovascular repair of abdominal aortic aneurysms: Where do we stand? *Mayo Clin. Proc.* **74**, 999–1010 (1999).

33. E. L. Chaikof, D. C. Brewster, R. L. Dalman, M. S. Makaroun, K. A. Illig, G. A. Sicard, C. H. Timaran, G. R. Upchurch Jr., F. J. Veith, The care of patients with an abdominal aortic aneurysm: The Society for Vascular Surgery practice guidelines. *J. Vasc. Surg.* **50**, S2–S49 (2009).

34. D. Mazzaccaro, L. Muzzarelli, A. Modafferi, G. Nano, Sizes of endografts for endovascular aortic repair: Do few fit most? *Ann. Ital. Chir.* **90**, 287–291 (2019).

35. H. Londero, G. Lev, H. Bertoni, E. Mendaro, O. Santaera, L. M. Riera, O. Mendiz, Safety and feasibility of balloon-expandable stent implantation for the treatment of type I endoleaks following endovascular aortic abdominal aneurysm repair. *EuroIntervention* **6**, 740–743 (2011).

36. G. B. Lima, E. R. Tenorio, G. B. Marcondes, M. A. Khasawneh, B. C. Mendes, R. R. DeMartino, F. Shuja, J. J. Colglazier, M. Kalra, G. S. Oderich, Outcomes of balloon-expandable versus self-expandable stent graft for endovascular repair of iliac aneurysms using iliac branch endoprosthesis. *J. Vasc. Surg.* **75**, 1616–1623.e2 (2022).

37. A. Kaladji, A. Dumenil, G. Mahé, M. Castro, A. Cardon, A. Lucas, P. Haigron, Safety and accuracy of endovascular aneurysm repair without pre-operative and intra-operative contrast agent. *Eur. J. Vasc. Endovasc. Surg.* **49**, 255–261 (2015).

38. M. Conti, F. Auricchio, M. De Beule, B. Verhegghe, Numerical simulation of Nitinol peripheral stents: From laser-cutting to deployment in a patient specific anatomy. *ESOMAT* **2009**, 06008 (2009).

39. E. L. Chaikof, D. C. Brewster, R. L. Dalman, M. S. Makaroun, K. A. Illig, G. A. Sicard, C. H. Timaran, G. R. Upchurch Jr., F. J. Veith, SVS practice guidelines for the care of patients with an abdominal aortic aneurysm: Executive summary. *J. Vasc. Surg.* **50**, 880–896 (2009).

40. A. Rivadeneyra, J. Fernández-Salmerón, J. Banqueri, J. A. López-Villanueva, L. F. Capitan-Vallvey, A. J. Palma, A novel electrode structure compared with interdigitated electrodes as capacitive sensor. *Sens. Actuators B* **204**, 552–560 (2014).

41. Y. Zhang, S. Xu, H. Fu, J. Lee, J. Su, K. C. Hwang, J. A. Rogers, Y. Huang, Buckling in serpentine microstructures and applications in elastomer-supported ultra-stretchable electronics with high areal coverage. *Soft Matter* **9**, 8062–8070 (2013).

42. E. Kissa, Capillary sorption in fibrous assemblies. *J. Colloid Interface Sci.* **83**, 265–272 (1981).

43. A. A. Nassr, W. H. Ahmed, W. W. El-Dakhakhni, Coplanar capacitance sensors for detecting water intrusion in composite structures. *Meas. Sci. Technol.* **19**, 075702 (2008).

44. A. Yazdani, G. E. Karniadakis, Sub-cellular modeling of platelet transport in blood flow through microchannels with constriction. *Soft Matter* **12**, 4339–4351 (2016).

45. Y. Asakura, A. Sapkota, O. Maruyama, R. Kosaka, T. Yamane, M. Takei, Relative permittivity measurement during the thrombus formation process using the dielectric relaxation method for various hematocrit values. *J. Artif. Organs* **18**, 346–353 (2015).

46. K. Tomaszek, F. Rahman, Case review and imaging: Treatment of central venous catheter associated fibrin sheath and chronic thrombus with the ClotTriever system. *Radiol. Case Rep.* **19**, 553–566 (2024).

47. J. N. Suojanen, D. P. Brophy, I. Nasser, Thrombus on indwelling central venous catheters: The histopathology of "fibrin sheaths". *Cardiovasc. Intervent. Radiol.* **23**, 194–197 (2000).

48. G. Mancia, A. Ferrari, L. Gregorini, G. Parati, G. Pomidossi, G. Bertinieri, G. Grassi, M. di Rienzo, A. Pedotti, A. Zanchetti, Blood pressure and heart rate variabilities in normotensive and hypertensive human beings. *Circ. Res.* **53**, 96–104 (1983).

49. S. Zhu, W. Dou, X. Zeng, X. Chen, Y. Gao, H. Liu, S. Li, Recent advances in the degradability and applications of tissue adhesives based on biodegradable polymers. *Int. J. Mol. Sci.* **25**, 5249 (2024).

50. D. F. Farrar, Bone adhesives for trauma surgery: A review of challenges and developments. *Int. J. Adhesion Adhes.* **33**, 89–97 (2012).

51. E. W. Washburn, The Dynamics of Capillary Flow. *Phys. Rev.* **17**, 273–283 (1921).

52. Y. Yuan, T. R. Lee, "Contact Angle and Wetting Properties" in *Surface Science Techniques* (Springer, 2013), pp. 3–34.

53. A. P. McGuigan, M. V. Sefton, Vascularized organoid engineered by modular assembly enables blood perfusion. *Proc. Natl. Acad. Sci. U.S.A.* **103**, 11461–11466 (2006).

54. G. Park, H. J. Chung, K. Kim, S. A. Lim, J. Kim, Y. S. Kim, Y. Liu, W. H. Yeo, R. H. Kim, S. S. Kim, J. S. Kim, Y. H. Jung, T. I. Kim, C. Yee, J. A. Rogers, K. M. Lee, Immunologic and tissue biocompatibility of flexible/stretchable electronics and optoelectronics. *Adv. Healthc. Mater.* **3**, 515–525 (2014).

55. P. J. Carreau, Rheological equations from molecular network theories. *Trans. Soc. Rheol.* **16**, 99–127 (1972).

56. W. J. Lee, S. Y. Park, H. J. Nam, S. H. Choa, Mechanical and optical characteristics of transparent stretchable hybrid substrate using PDMS and ecoflex material. *J. Microelectron. Packag. Soc.* **25**, 129–135 (2018).

57. C. G. Caro, T. J. Pedley, R. C. Schroter, W. A. Seed, *The Mechanics of the Circulation* (Cambridge Univ. Press, ed. 2, 2012).

58. Y.-J. Hong, H. Jeong, K. W. Cho, N. Lu, D.-H. Kim, Wearable and implantable devices for cardiovascular healthcare: From monitoring to therapy based on flexible and stretchable electronics. *Adv. Funct. Mater.* **29**, 1808247 (2019).

59. Q.-A. Huang, L. Dong, L.-F. Wang, LC passive wireless sensors toward a wireless sensing platform: Status, prospects, and challenges. *J. Microelectromechanical Syst.* **25**, 822–841 (2016).

60. S. E. J. Connor, A. Bleetman, M. J. Duddy, Safety standards for stab-resistant body armour: A computer tomographic assessment of organ to skin distances. *Injury* **29**, 297–299 (1998).

61. L. W. Bartels, H. F. Smits, C. J. Bakker, M. A. Viergever, MR imaging of vascular stents: Effects of susceptibility, flow, and radiofrequency eddy currents. *J. Vasc. Interv. Radiol.* **12**, 365–371 (2001).

62. J. P. K. Sampath, A. Alphones, H. Shimasaki, "Coil design guidelines for high efficiency of wireless power transfer (WPT)" in *2016 IEEE Region 10 Conference* (*TENCON*) (IEEE, 2016), pp. 726–729.

63. B. H. Waters, B. J. Mahoney, G. Lee, J. R. Smith, “Optimal coil size ratios for wireless power transfer applications” in *2014 IEEE International Symposium on Circuits and Systems* (*ISCAS*) (IEEE, 2014), pp. 2045–2048.

64. W. H. Ko, S. P. Liang, C. D. F. Fung, Design of radio-frequency powered coils for implant instruments. *Med. Biol. Eng. Comput.* **15**, 634–640 (1977).

65. M. Soma, D. C. Galbraith, R. L. White, Radio-frequency coils in implantable devices: Misalignment analysis and design procedure. *IEEE Trans. Biomed. Eng.* **34**, 276–282 (1987).

66. J. O. Mur-Miranda, G. Fanti, Y. Feng, K. Omanakuttan, R. Ongie, A. Setjoadi, N. Sharpe, “Wireless power transfer using weakly coupled magnetostatic resonators” in *2010 IEEE Energy Conversion Congress and Exposition* (IEEE, 2010), pp. 4179–4186.

67. C. Marchal, M. Nadi, A. J. Tosser, C. Roussey, M. L. Gaulard, Dielectric properties of gelatine phantoms used for simulations of biological tissues between 10 and 50 MHz. *Int. J. Hyperthermia* **5**, 725–732 (1989).

68. E. Knöps, J. van Schaik, K. E. A. van der Bogt, H. T. C. Veger, H. Putter, E. J. Waasdorp, J. R. van der Vorst, Stent graft sizing for endovascular abdominal aneurysm repair using open source image processing software. *Ann. Vasc. Surg.* **71**, 411–418 (2021).

69. M. W. Siebert, P. S. Fodor, Newtonian and non-Newtonian blood flow over a backward-facing step—A case study, paper presented at the COMSOL Conference 2009, Boston, MA, 2009.

70. F. M. White, *Fluid Mechanics* (McGraw-Hill, ed. 5, 2003), vol. 3. McGraw-Hill series in mechanical engineering.

71. S. Yadav, S. J. Shire, D. S. Kalonia, Viscosity analysis of high concentration bovine serum albumin aqueous solutions. *Pharm. Res.* **28**, 1973–1983 (2011).
